# Oral Tolerance Induced by Transfer of Food Antigens via Breast Milk of Allergic Mothers Prevents Offspring from Developing Allergic Symptoms in a Mouse Food Allergy Model

**DOI:** 10.1155/2012/721085

**Published:** 2012-03-27

**Authors:** Takeshi Yamamoto, Yuma Tsubota, Toshihisa Kodama, Natsuko Kageyama-Yahara, Makoto Kadowaki

**Affiliations:** Division of Gastrointestinal Pathophysiology, Institute of Natural Medicine, University of Toyama, 2630 Sugitani, Toyama 930-0194, Japan

## Abstract

We examined whether maternal exposure to food antigens during lactation and maternal allergic status would affect the development of food allergy in offspring. OVA-sensitized or OVA-nonsensitized BALB/c female mice were exposed or unexposed to OVA during lactation. After weaning, their offspring were systemically sensitized twice with OVA and repeatedly given OVA by oral intubation. While 97.1% of the mice breastfed by OVA-nonsensitized and OVA-unexposed mothers developed allergic diarrhea, 59.7% of the mice breastfed by OVA-exposed nonallergic mothers during lactation and 24.6% of the mice breastfed by OVA-exposed allergic mothers during lactation developed food allergy. Furthermore, OVA was detected in breast-milk from OVA-exposed nonallergic mothers during lactation (4.6 ± 0.5 *μ*g/mL). In addition, OVA-specific IgG1 titers were markedly increased in breast milk from allergic mothers (OVA-sensitized and OVA-unexposed mother: 11.0 ± 0.5, OVA-sensitized and OVA-exposed mother: 12.3 ± 0.3). Our results suggest that oral tolerance induced by breast milk-mediated transfer of dietary antigens along with their specific immunoglobulins to offspring leads to antigen-specific protection from food allergy.

## 1. Introduction

Over the last few decades, the prevalence of allergic diseases, such as atopic dermatitis, bronchial asthma allergic rhinitis, and food allergy (FA), has dramatically increased in advanced countries. Since FA is relatively common in the early stage of the “Allergy March”, in which symptoms are exhibited successively with age, it is very important for infants to outgrow their FA from a viewpoint of primary prevention of various allergy diseases [1, 2]. However, until now, there have not been any effective drug therapies for FA.

The intestinal epithelium is constantly exposed to a multitude of foreign materials that are either harmful or beneficial for humans. Consequently, the intestinal immune system must balance between protective immune responses that are induced by encounters with intestinal pathogens and tolerance against commensal bacteria and food antigens. Despite the large extent of dietary antigenic exposure, the intestinal mucosal immune system has the unique propensity to evoke tolerance against orally administrated antigens and thereby maintains optimal immunological homeostasis in the intestine. It has been assumed that a breakdown in oral tolerance mechanisms or a failure in the induction of oral tolerance results in allergic food hypersensitivity [2–4].

Previous studies have shown that breast milk can influence the incidence of allergic diseases in infants. Epidemiological studies on the protection from allergic diseases caused by breastfeeding have yielded conflicting results. Saarinen and Kajosaari concluded from a prospective study in patients up to 17 years old that the incidence of allergic diseases was lower in the group of patients who were fed by breast milk for a long period [5]. On the contrary, it has been reported that complete breastfeeding for several months postnatally cannot protect children against allergic diseases [6]. For many years, the American Academy of Pediatricians and the United Kingdom government have recommended maternal food antigen avoidance during pregnancy and lactation to reduce FA. Actually, it is known that there are sufficient concentrations of food antigens for making infants develop FA [7]. However, until now, there have not been any conclusive data that maternal food antigen restriction is protective against FA in infants [8].

Furthermore, it remains controversial whether the mother's allergic status is a significant risk factor for the development of allergy in breastfed infants. In recent prospective studies on a birth cohort of 3,115 children, breast feeding was demonstrated to be associated with a lower asthma risk in children up to 8 years of age without evidence of attenuation of the association and regardless of the child's family history of allergy [9].

It remains unclear whether maternal exposure to food antigens during lactation and maternal allergic status affects the development of allergic diseases in offspring via transfer of factors influencing susceptibility or resistance to allergic diseases. Therefore, we investigated the ability of breast milk from allergic mothers exposed to food antigens during lactation to protect breastfed offspring from FA.

## 2. Methods

### 2.1. Animals

BALB/c mice (Japan SLC, Shizuoka, Japan) were used for the experiments and housed in the experimental animal facility at University of Toyama. This study was approved by the Animal Experiment Committee in University of Toyama (Authorization No. is S-2009 INM-9) in accordance with the Guide for the Care and Use of Laboratory Animals in University of Toyama which is accredited by the Ministry of Education, Culture, Sports, Science, and Technology, Japan.

### 2.2. Experimental Protocols

**Figure 1:**
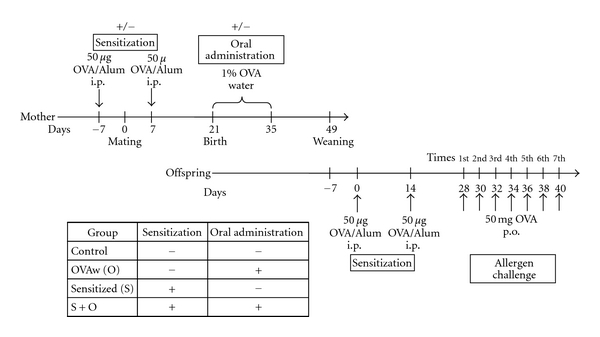
Experimental protocol. Sensitized mice were exposed to 1% OVA in drinking water for 2 weeks immediately after delivery. Offspring were weaned at 4 weeks and 5-week-old offspring were used for the FA model.

To study the relationship between breastfeeding and allergic diseases, 9-week-old female BALB/c mice were sensitized twice at a 2-week interval with 50 *μ*g of OVA in the presence of 1.3 mg of aluminum hydroxide gel by intraperitoneal injection. Nonsensitized (vehicle-injected) and sensitized mice were mated with naïve males 7 days after the first intraperitoneal OVA injection. Subsequently, lactating mice were exposed to 1% OVA in drinking water for 2 weeks immediately after delivery (the duration of pregnancy in BALB/c mice is about 21 days). The offspring were weaned at 4 weeks, and 5-week-old offspring were used to test our FA model (Figure 1). Brief, BALB/c mice were sensitized twice at a 2-week interval with 50 *μ*g of OVA in the presence of 1.3 mg of aluminum hydroxide gel (Sigma-Aldrich), which served as an adjuvant, by intraperitoneal injection. Two weeks after systemic priming, the mice were repeatedly given 50 mg of OVA using intragastric feeding needles three times per week. Allergic diarrhea was assessed by visually monitoring mice up to 1 hour following intragastric OVA challenge. Mice with profuse liquid stool were recorded as allergic diarrhea-positive animals. Tissues and plasma were obtained 1hour after the seventh oral OVA challenge.

### 2.3. OVA-Specific Immunoglobulin Level in Plasma and Breast Milk

OVA-specific IgE, IgA, and IgG1 levels in plasma as well as OVA-specific IgA and IgG1 levels in breast milk were measured by the sandwich ELISA method. The sensitive sandwich ELISAs used to analyze OVA-specific IgE, IgA, and IgG1 levels were developed using biotinylated rat anti-mouse IgE (1 : 1,000; YAMASA, Tokyo, Japan), HRP-conjugated sheep anti-mouse IgA (1 : 2,000; SouthernBiotech, Birmingham, AL, USA), and HRP-conjugated sheep anti-mouse IgG1 (1 : 2,000; SouthernBiotech). Plates were coated with 10 *μ*g/mL OVA (Fraction V; Sigma-Aldrich) at 4°C overnight and blocked with PBS containing 2% Block Ace (DS Pharma Biomedical, Osaka, Japan). Diluted samples were added to the plates, which were then incubated for 2 hour at 37°C. Each HRP-conjugated anti-immunoglobulin antibody was added and the plates were then incubated for 1 hour at 37°C. Reactions were developed by 3,3′,5,5′-tetramethylbenzidine (Sigma-Aldrich), and color development was terminated by 0.5 N HCl. Endpoint titers of OVA-specific immunoglobulin antibodies were expressed as the reciprocal log_2_ of the last dilution that showed a level of >0.1 absorbance over the background levels, which gave an absorbance at 450 nm. Breast milk was collected from the stomachs of 7-day-old neonatal mice and diluted 10 times with RPMI1640 medium. The diluted breast milk was centrifuged, and the supernatant was collected as a breast milk sample.

### 2.4. Measurement of OVA Concentration in Breast Milk

OVA levels in breast milk were analyzed using ITEA OVA ELISA Kit (Precoated) (ITEA, Tokyo, Japan). Measurements were performed according to the manufacturer's instructions.

### 2.5. Expression of mRNA in the Intestine

mRNA expression levels in the intestine were examined according to the method previously described [10]. Briefly, 1 hour after the seventh oral OVA challenge, 2 cm of the mouse proximal colon was excised. Total RNA was extracted from the proximal colon using Sepasol Super (Nacalai Tesque, Kyoto, Japan) according to the manufacturer's instruction. Reverse transcription was performed using the ExScript RT reagent Kit (Takara Bio, Shiga, Japan) and random primers, followed by real-time PCR. Real-time PCR amplification of IL-4, IFN-*γ*, Foxp3, and GAPDH was performed using SYBR Premix Ex Taq (Takara Bio). Target mRNA expression was normalized to GAPDH mRNA expression as an internal control in each sample. The results were expressed as the relative ratio to the naïve group average.

### 2.6. Immunohistochemistry on Mucosal Mast Cells in the Intestine

Immunohistochemistry was performed according to the method previously described [10]. Briefly, the excised proximal colon was fixed in 4% paraformaldehyde (w/v) in 0.1 M sodium phosphate buffer (PB, pH 7.3) at 4°C for 12–18 hour. Frozen sections (30 *μ*m) were cut at −20°C using a cryostat microtome (Leica Microsystems, Nussloch, Germany). The sections were exposed for 12–18 hour to antiserum against mouse mast cell protease-1 (mMCP-1; a marker of mouse mucosal mast cells; 1 : 5000, Moredun Scientific, Scotland, UK), washed with 0.01 M PBS and incubated for 2 hour with Cy3-conjugated sheep anti-donkey IgG (1 : 200, Jackson Immunoresearch Laboratories, West Grove, PA, USA). The immunostained sections were examined using a fluorescence microscope (IX71 System, Olympus, Tokyo, Japan) with the filter set U-MWIG3 (Olympus) and photographed using an Olympus digital camera (DP70, Olympus). The brightness and contrast of the images were modified with Adobe Photoshop Elements 2.0 (Adobe Systems, San Jose, CA, USA).

### 2.7. Data Analyses

The data are expressed as the means ± S.E. or the means and dot plots. Statistical comparisons were made using two-tailed Student's unpaired *t*-tests or one-way ANOVA followed by Tukey's post-hoc test for multiple comparisons of data with a normal distribution or Games-Howell test for multiple comparisons of data with a nonnormal distribution. *P* values less than 0.05 were considered to be statistically significant.

## 3. Results

### 3.1. Breast Milk from OVA-Exposed Allergic Mothers during Lactation Protects Offspring from Allergic Symptoms in Food Allergy Model

**Figure 2:**
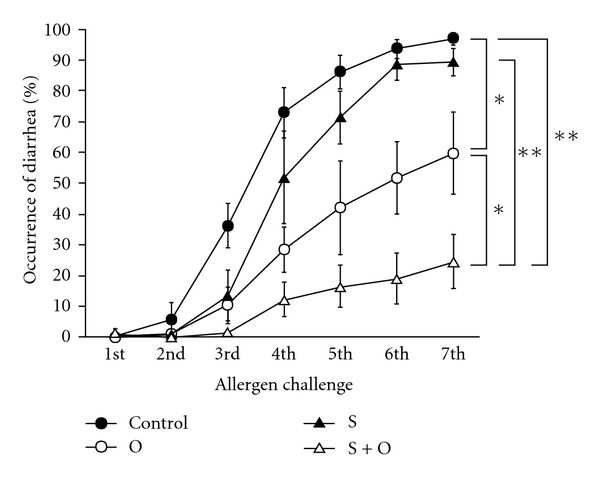
Effects of the maternal exposure to food antigens during lactation and the maternal allergic status on allergic symptoms in FA model. Offspring breastfed by OVA-sensitized and OVA-exposed lactating mothers were more protected from the development of allergic symptoms of FA compared with offspring from OVA-nonsensitized and OVA-exposed lactating mothers. Data are shown as the means ± SE (8 independent experiments, total *n* = 62–181). *P < 0.05, **P < 0.01.

Offspring breastfed by OVA-nonsensitized and OVA-unexposed mothers developed the symptoms of FA (Figure 2: Control group: 97.1 ± 1.9% after the seventh oral OVA challenge). We next determined whether maternal exposure to 1% OVA in drinking water during lactation affects intestinal allergic symptoms in offspring. We found that about half of the offspring breastfed by OVA-nonsensitized and OVA-exposed mothers exhibited allergic reactions after the seventh OVA challenge (Figure 2: O group; 59.7 ± 13.2% after the seventh oral OVA challenge, *P* < 0.05 compared with the control group). Furthermore, when compared with the O group offspring, a reduction in allergic symptoms was evident in offspring nursed by OVA-sensitized and OVA-exposed mothers (Figure 2: S + O group; 24.6 ± 8.8% after the seventh oral OVA challenge, *P* < 0.05 compared with the O group), although breast milk from OVA-sensitized and OVA-unexposed mothers had little effect on the development of FA in offspring (Figure 2: S group: 89.7 ± 4.4% after the seventh oral OVA challenge).

**Figure 3:**
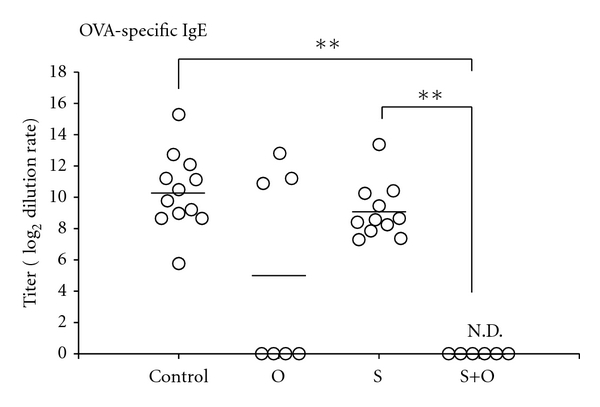
Effects of the maternal exposure to food antigens during lactation and the maternal allergic status on plasma IgE levels of offspring in the mouse FA model. Plasma IgE levels were undetectable in offspring breastfed by OVA-sensitized and OVA-exposed lactating mothers. Data are shown as the means and individual data points. N.D.: not detectable. **P < 0.01, *n* = 6–11.

Plasma levels of OVA-specific IgE was very high in the control group offspring (Figure 3). OVA-specific IgE in S + O group offspring were virtually undetectable (Figure 3: *P* < 0.01 compared with the control group), while S group offspring exhibited high OVA-specific IgE levels comparable with the control group offspring (Figure 3). Although OVA-specific IgE levels in O group offspring tended to be lower compared with those in the control group offspring, there were large individual differences among the O group offspring (Figure 3).

### 3.2. Maternal Exposure to OVA during Lactation Suppresses the Th2-Polarized Cytokine Profile in the Proximal Colons of Offspring with Food Allergy

We examined Th1 (IFN-*γ*) and Th2 (IL-4) cytokine profiles in the proximal colons of the offspring in each of the four groups. IFN-*γ* mRNA expression in the proximal colon was not enhanced in the FA model mice and not affected by the maternal exposure to OVA during lactation and/or the maternal allergic status.

**Figure 4:**
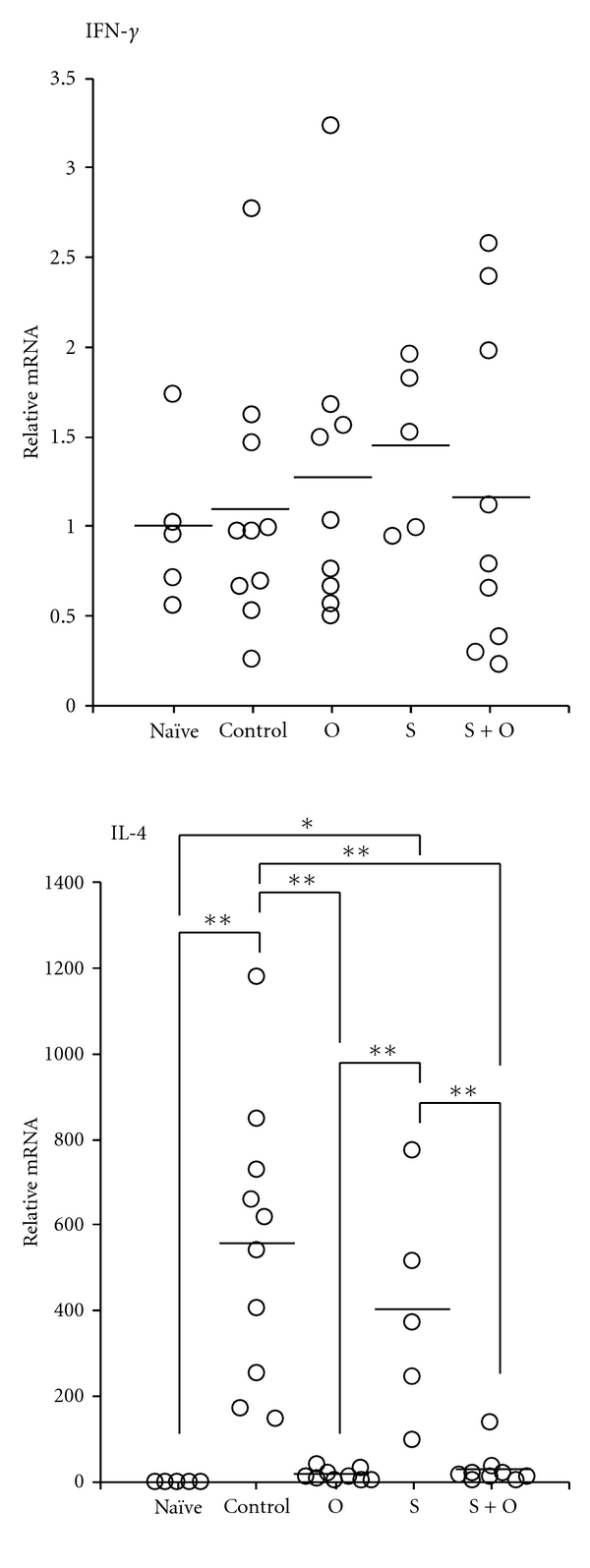
Effects of the maternal exposure to food antigens during lactation and the maternal allergic status on Th1 and Th2 cytokine profiles in the proximal colons of offspring with FA. The mRNA expression levels of IL-4 were completely reduced to the naïve mice range in offspring breastfed by OVA-exposed mothers during lactation, but IFN-*γ* mRNA expression levels were not altered by the maternal exposure to food antigens and the maternal allergic status. Data are shown as the means and individual data points. *P < 0.05, **P < 0.01, *n* = 5–10.

Conversely, IL-4 mRNA expression was greatly upregulated in the proximal colons of the control group offspring (Figure 4: *P* < 0.01, 556.4 ± 102.6 compared with naïve mice: 1.0 ± 0.2), and the maternal exposure to OVA during lactation markedly prevented the enhancement of IL-4 mRNA expression in the proximal colons of both the O group and S + O group offspring (Figure 4: *P* < 0.01, 16.4 ± 4.8 and 30.6 ± 14.2, respectively, compared with the control group offspring). The S group offspring exhibited high levels of IL-4 mRNA expression that were comparable to those of the control group offspring (Figure 4: 402.3 ± 116.1).

### 3.3. Maternal Exposure to OVA during Lactation Prevents Mucosal Mast Cell Infiltration in the Proximal Colons of Offspring with Food Allergy

**Figure 5:**
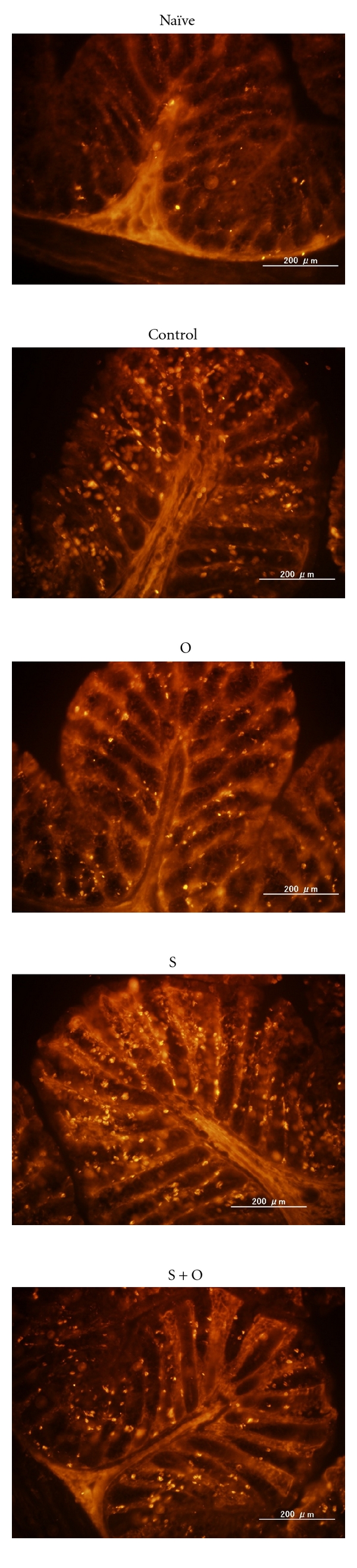
Effects of the maternal exposure to food antigens during lactation and the maternal allergic status on mucosal mast cell infiltration in the proximal colons of offspring FA. The infiltration of mucosal mast cells was greatly attenuated in offspring with FA breastfed by OVA-exposed mothers during lactation.

Mucosal mast cells were dramatically increased in the proximal colons of the control group offspring, while they were markedly decreased in the proximal colons of both the O group and S + O group offspring. Many mucosal mast cells were observed in the proximal colons of the S group offspring, and these levels were comparable to those in the proximal colons of the control group offspring (Figure 5).

### 3.4. Maternal Transfer of OVA and/or OVA-Specific Antibodies Through Breast Milk

**Figure 6:**
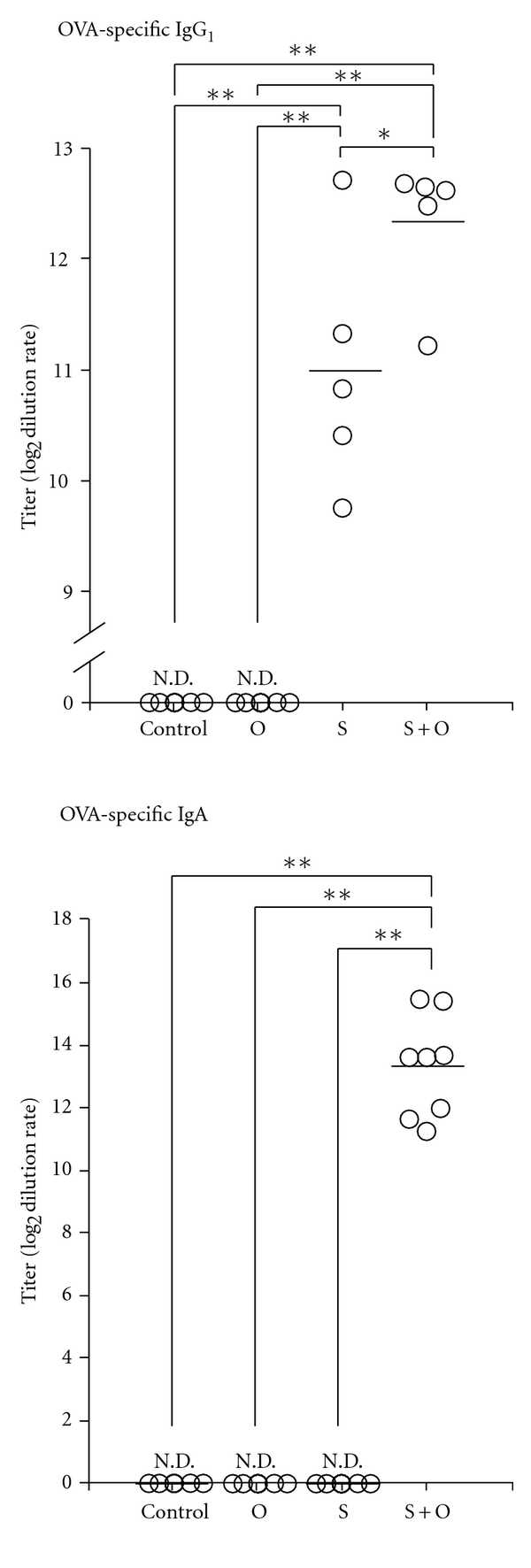
Effects of the maternal exposure to food antigens during lactation and the maternal allergic status on OVA-specific IgG1 and IgA levels in breast milk. We found OVA-specific IgG1 only in the breast milk from OVA-sensitized mothers, which implies that mother mice secrete IgG1 into their breast milk. We found OVA-specific IgA only in the breast milk from OVA-sensitized and OVA-exposed mothers. Data are shown as the means and individual data points. N.D.: not detectable. *P < 0.05, **P < 0.01, *n* = 5–9.

Next, we determined whether the allergic symptoms in offspring were associated with maternal transfer of OVA and/or OVA-specific antibodies (IgG1 and IgA) through breast milk. Among the mothers of the four groups, OVA was only detected in the breast milk of mothers from the O group (4.6 ± 0.5 *μ*g/mL, *n* = 5). Although OVA-specific IgG1 were not detected in the breast milk from the control and O group mothers, the sensitization of mothers to OVA increased the concentration of OVA-specific IgG1 in the breast milk of the S + O group mothers (S group mother: 11.0 ± 0.5, S + O group mother: 12.3 ± 0.3; Figure 6, *P* < 0.01, *n* = 5). In contrast, OVA-specific IgA was detected only in the breast milk from the S + O group mothers (Figure 6, *n* = 5–9).

**Figure 7:**
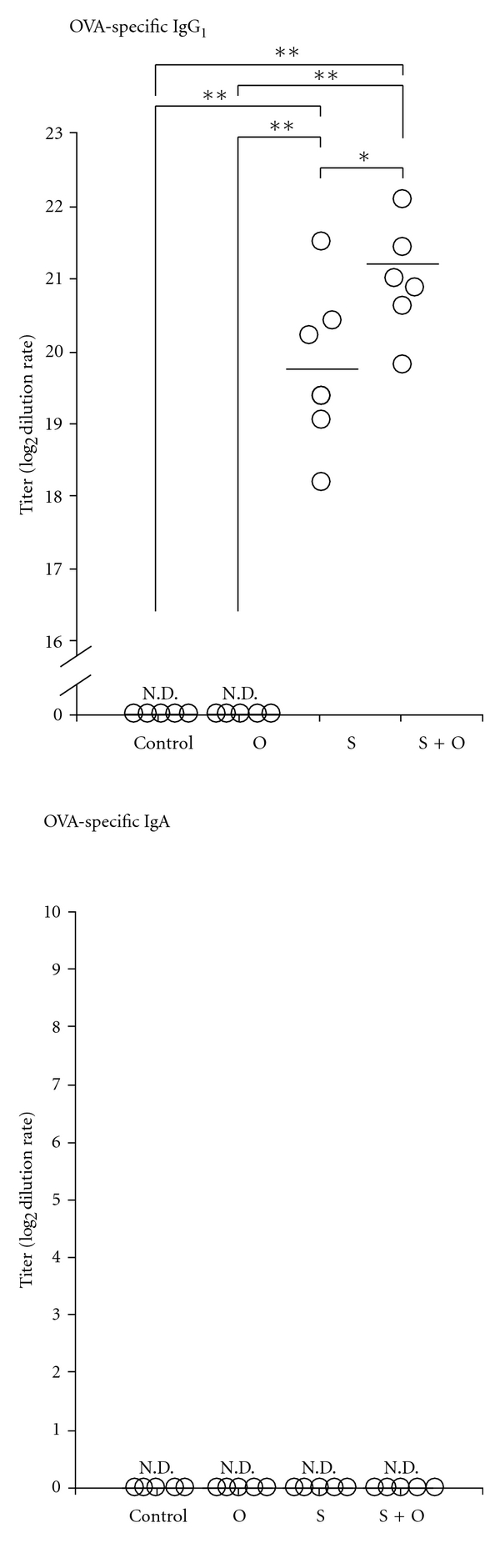
Effects of the maternal exposure to food antigens during lactation and the maternal allergic status on plasma OVA-specific IgG1 and IgA levels in offspring. We found OVA-specific IgG1 only in the plasma of naïve offspring breastfed by OVA-sensitized mothers, which implies that OVA-specific IgG1 transferred from mothers circulate in their offspring. Plasma OVA-specific IgA levels were undetectable in the four groups of offspring. Data are shown as the means and individual data points. N.D.: not detectable. *P < 0.05, **P < 0.01, *n* = 5–7.

We further analyzed plasma OVA-specific IgG1 and IgA levels in 7-days-old offspring of the four groups to elucidate the transfer of OVA-specific IgG1 and IgA from mothers to offspring via breast milk. Similarly to OVA-specific IgG1 in the breast milk, plasma OVA-specific IgG1 was detected in both the S group and S + O group offspring but not in the control group and O group offspring (Figure 7). Conversely, plasma OVA-specific IgA was undetectable in the offspring of all four groups (Figure 7).

## 4. Discussion

This study was performed to test our hypothesis that maternal factors transferred through breast milk affect the development of FA in offspring. In the present study, using a mouse FA model, we demonstrated that the induction of oral tolerance can suppress food allergic symptoms and that maternal ingestion of food antigens during lactation, especially in allergic mothers, can induce effective oral tolerance, thereby preventing the onset of FA.

### 4.1. Induction of Oral Tolerance

Early tolerance induction is an attractive approach for primary prevention of allergic diseases. Recently, the preventive effects of oral tolerance on allergic diseases such as allergic asthma and allergic rhinitis have been reported in experimental models [11–13].

Experimental FA models where repeated oral challenges with OVA in systemically OVA-primed BALB/c mice led to allergic diarrhea require aberrant Th2-type responses, such as an enhanced production of IL-4, IL-5, and IL-13 by the spleen and large intestine and high levels of OVA-specific plasma IgE [14, 15]. We have reported that phosphatidylinositol-3 kinase deficient mice (BALB/c background) selectively lacking gastrointestinal mast cells could not develop allergic diarrhea in our FA model [16], suggesting that mucosal mast cells in the intestine play an important role in the pathogenesis and development of allergic diarrhea.

In 2011, Hadis et al. have demonstrated that the induction of oral tolerance prevents food allergic diarrhea in adult mice [17]. Here we also show that oral tolerance induced by the exposure to OVA prevents the increase in plasma OVA-specific IgE levels and Th2 cytokine mRNA expression in both systemic and mucosal immune system, in addition, the exposure to OVA abrogates the augmentation of the infiltration of mucosal mast cells into the colon (see supplemental materials available at doi:10.1155/2012/721085, Supplemental Figures 1–5).

### 4.2. Influence of the Maternal Exposure to Food Antigens during Lactation on Allergic Symptoms

Interestingly, Verhasselt et al. demonstrated that breast-milk-mediated transfer of antigens to the neonate results in oral tolerance induction leading to antigen-specific protection from allergic airway disease in a mouse experimental model [18]. However, it remains controversial in humans whether food antigen ingestion by the lactating mother affects the development of FA in neonates. Thus, in the current study, we addressed this issue with our mouse FA model. We found that breastfeeding by OVA-nonsensitized and OVA-exposed mothers moderately reduced the incidence of allergic symptoms in neonates, and food antigen OVA was detected in breast milk only from OVA-nonsensitized and OVA-exposed mothers during lactation, indicating that the prior neonatal exposure to the low levels of food antigens within the breast milk from these mothers for 2 weeks moderately alleviates the susceptibility of their neonates to FA. Furthermore, our results indicate that OVA transferred through breast milk from OVA-exposed mothers during lactation induces oral tolerance, which suppresses the increases in plasma OVA-specific IgE levels and Th2 cytokine mRNA expression levels in the mucosal immune system and the augmentation of the infiltration of mucosal mast cells into the colon, thereby alleviating the development of FA. Food antigens ingested by mothers during lactation are also secreted into breast milk in humans [7, 19]. Taken together, the present results led us to propose the hypothesis that induction of oral tolerance by the maternal ingestion of food antigens during lactation is a strategy for the prevention of FA in infants. However, breast milk from OVA-nonsensitized and OVA-exposed mothers during lactation protects only about half of the offspring from allergic symptoms and drastic increases in plasma OVA-specific IgE levels, implying that the induction of oral tolerance simply by the maternal exposure to OVA during lactation is not sufficient for the prevention of FA in offspring.

### 4.3. Influence of the Mother's Allergic Status on the Susceptibility of Offspring to Food Allergy

Mosconi et al. demonstrated that breastfeeding by antigen-exposed sensitized mothers abolishes asthma development in progeny. Compared with the protection elicited by antigen-exposed nonsensitized mothers, protection by sensitized mothers was much more profound [20]. Furthermore, López-Expósito et al. showed that low-dose food antigen exposure during pregnancy and lactation reduces the risk of offspring developing first-exposure food antigen-induced anaphylaxis and specific IgE production to active food antigen sensitization in offspring in a mouse peanut FA model [21]. In the present study, we showed that offspring breastfed by OVA-sensitized and OVA-exposed mothers were dramatically protected from the development of FA. Furthermore, OVA-specific IgA and IgG1 were detected in the breast milk from these mothers. In contrast, breast milk from OVA-sensitized and OVA-unexposed mothers had little effect on the development of FA in offspring.

It is known that antigen-specific IgA and IgG form immune complexes with antigens in breast milk [20, 22, 23]. Therefore, it is assumed that OVA that has been ingested by allergic mothers during lactation and then secreted into their breast milk immediately forms immunity complexes with maternal OVA-specific immunoglobulins, thereby masking epitopes of OVA detected by OVA-specific antibodies of OVA ELISA Kit. Thus, we could not measure OVA in the breast milk from OVA-sensitized and OVA-exposed mothers using OVA ELISA kit, while we could detect OVA in breast milk from OVA-nonsensitized and OVA-exposed mothers. It has been reported that an antigen-IgA immune complexes are efficiently taken up by the body by transcytosis as an immune complex through specific receptors on M cells [24–26].

Moreover, the neonatal Fc receptor for IgG (FcRn), which is the IgG receptor that is expressed on intestinal epithelial cells, is known to contribute to the absorption of IgG and antigen-IgG immune complexes within breast milk [27–29]. Mosconi et al. demonstrated that milk-borne OVA-IgG complexes are actively transferred from mothers to offspring by the FcRn. Furthermore, FcRn-mediated transfer of OVA-IgG complexes resulted in the induction of FoxP3^+^ regulatory T cells in mesenteric lymph node and that FcRn-deficient mice breastfed by OVA-exposed allergic mice were not protected from allergic airway disease [20]. In this study, we showed that OVA-specific IgG1 was detected in breast milk from allergic mothers, and OVA-specific IgG1 was also detected in the plasma of their offspring, while OVA-specific IgA was not detected in plasma of any offspring. Taken together, these data imply that OVA is effectively transferred into offspring via immune complexes of OVA and OVA specific IgG1 in breast milk through FcRn in comparison to OVA alone in breast milk and thereby effectively induces oral tolerance to protect neonates from FA.

## 5. Conclusions

Our results indicate that oral tolerance induced by breast-milk-mediated transfer of food antigens by specific immunoglobulins to offspring leads to antigen-specific protection from FA. Our findings may pave the way for the development of novel approaches to primary prevention of allergic diseases such as FA. Furthermore, pioneering researches should be undertaken to target the treatment with immune complexes of food antigen and food antigen-specific IgG1 for the effective prevention strategies against FA.

## Supplementary Material

Supplemental Figure 1: Experimental protocol. Method of FA induction and guidance method of oral tolerance in BALB/c mice.
Supplemental Figure 2: Occurrence of allergic diarrhea and effects of prior OVA exposure to induction of FA in BALB/c mice Repeated oral OVA (50 mg) challenges resulted in allergic diarrhea in the FA group (■). The occurrence of OVA-induced diarrhea was completely suppressed by prior exposure to 1% OVA in the drinking water for 5 days in the OT–FA group (□). Data are shown as the means ± SE (4 independent experiments, total n = 47–52). ∗∗ p < 0.01 compared with the FA group.
Supplemental Figure 3: Effects of the induction of oral tolerance on OVA–specific IgE in the plasma of mice with FA. OVA–specific IgE titers in plasma were markedly increased in FA mice, whereas OVA-specific IgE titers were undetectable in the plasma of OT–FA mice. Data are shown as the means and individual data points. N.D.= not detectable. *++*p < 0.01 compared with naïve mice, ∗∗p < 0.01 compared with FA mice, n = 3-4.
Supplemental Figure 4: Effects of the induction of oral tolerance on the mRNA expression levels of Th1 and Th2 cytokines in the proximal colons of mice with FA. Th2 cytokine (IL–4) mRNA was significantly up–regulated in FA mice, whereas prior OVA exposure to induction of FA completely prevented the enhanced expression levels of these cytokines. In contrast, the mRNA expression levels of Th1 cytokines (IFN–*γ*) were nearly identical among the three mice groups. Data are shown as the means and individual data points. ∗∗p < 0.01 compared with naive mice, *++*p < 0.01 compared with FA mice, n = 5.
Supplemental Figure 5: Effects of the induction of oral tolerance on mMCP–1–positive mucosal mast cells in the proximal colon. The number of mMCP–1 positive mucosal mast cells increased in FA mice. In contrast, OT–FA mice had few mMCP–1 positive mucosal mast cells in their proximal colons. Scale bar = 200 *μ*m.
