# Supplementary material for: Oral Tolerance Induced by Transfer of Food Antigens via Breast Milk of Allergic Mothers Prevents Offspring from Developing Allergic Symptoms in a Mouse Food Allergy Model
Source: Clin Dev Immunol. 2012 Mar 27;2012:721085. doi: 10.1155/2012/721085 (PMC3310277; doi:10.1155/2012/721085)

Supplemental Figure 1

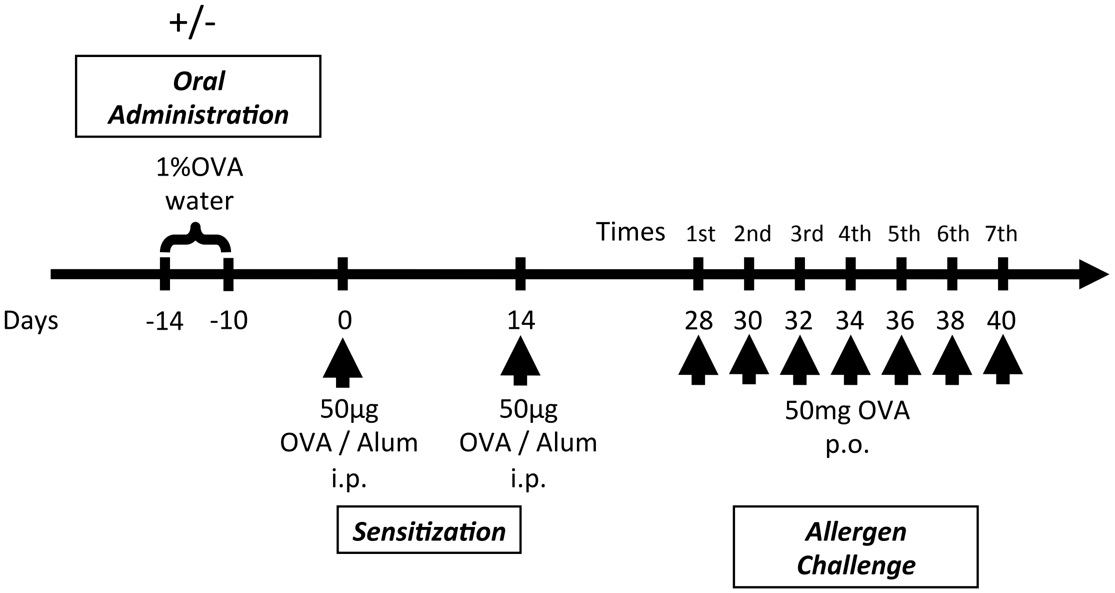

Supplemental Figure 2

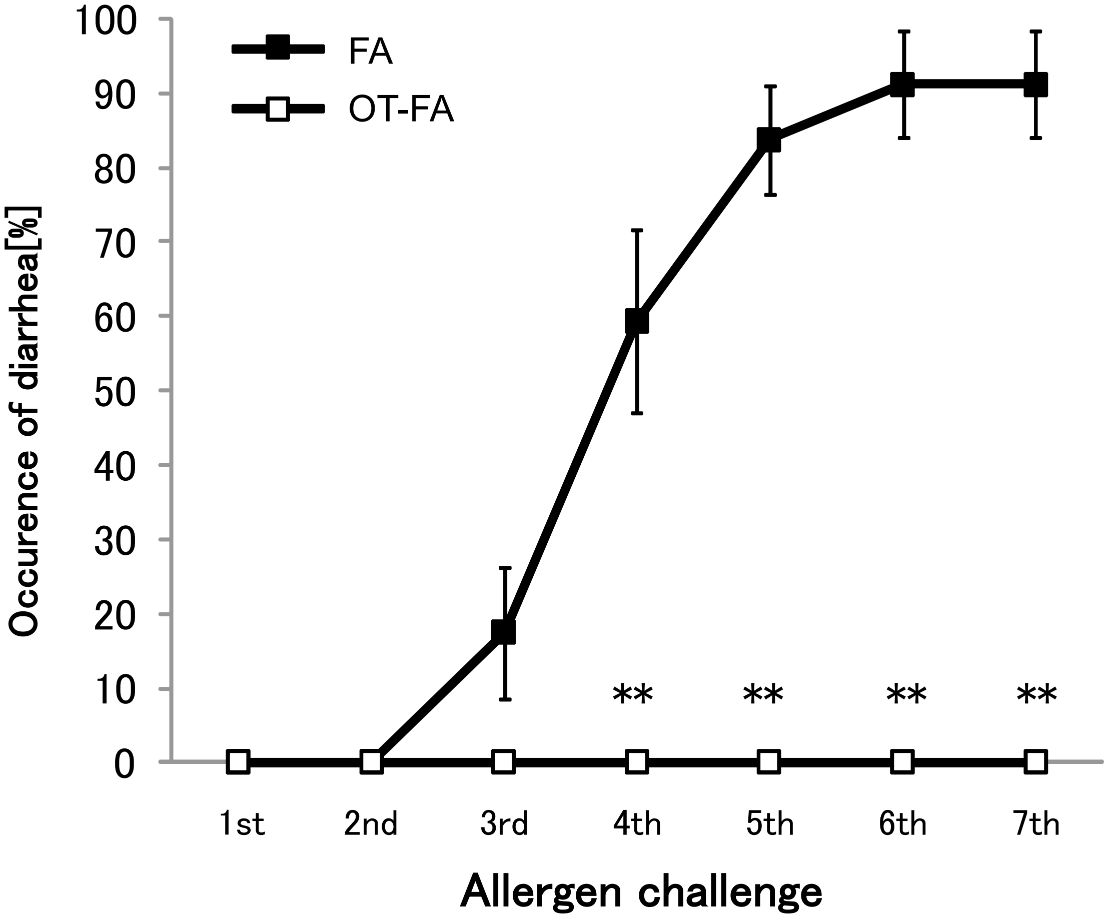

Supplemental Figure 3

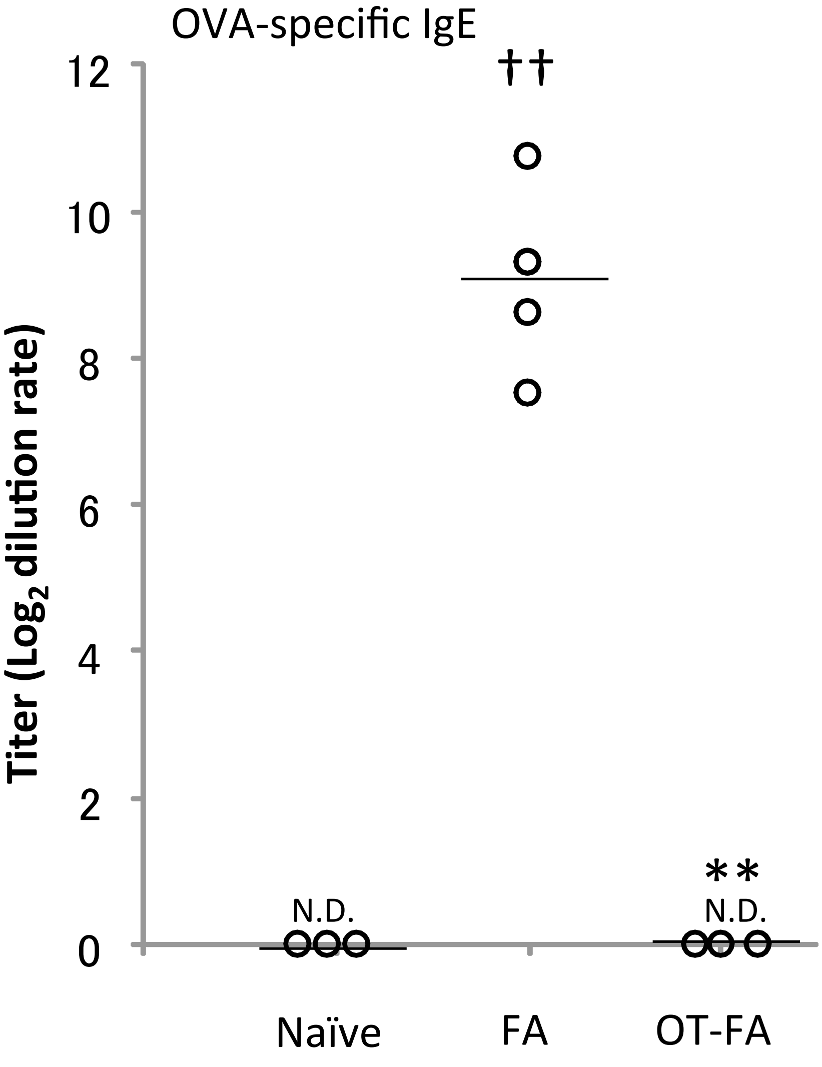

Supplemental Figure 4

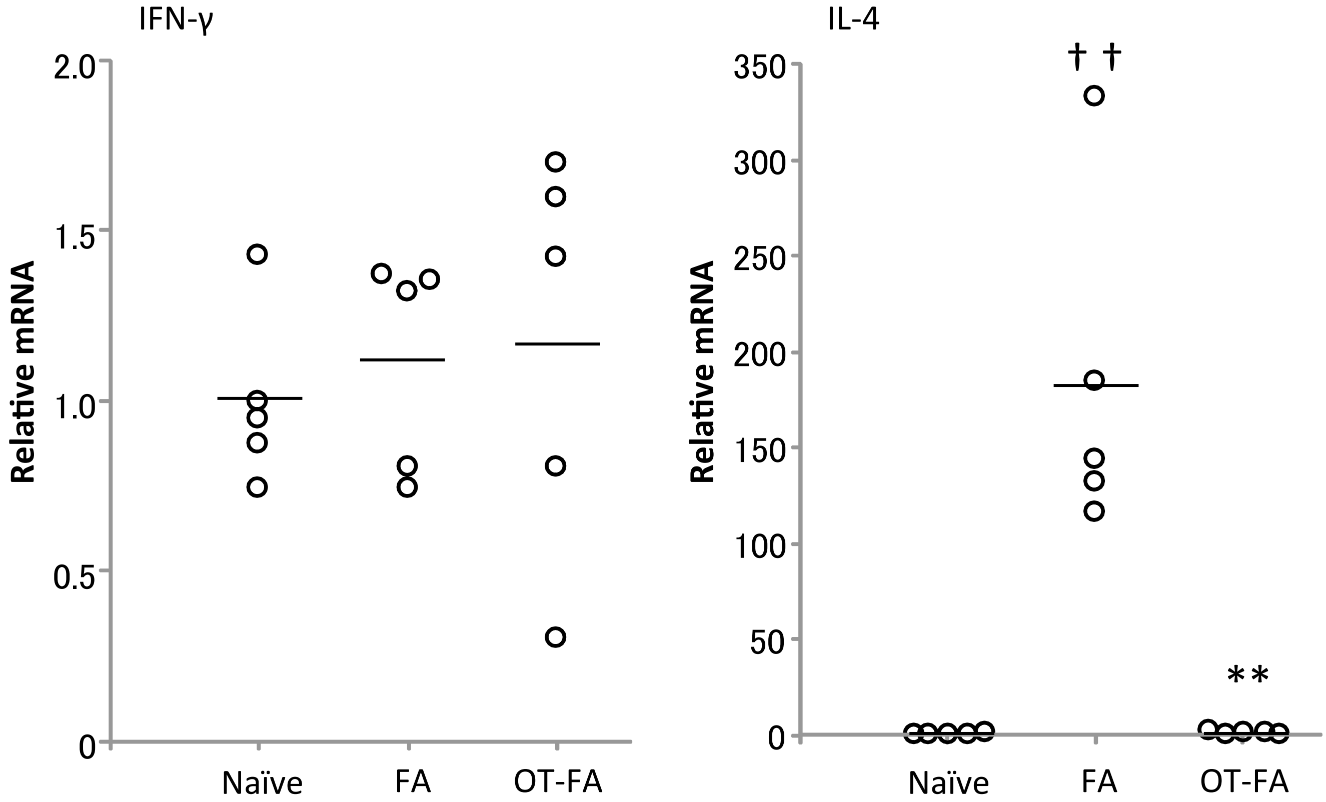

Supplemental Figure 5

Naïve FA OT-FA

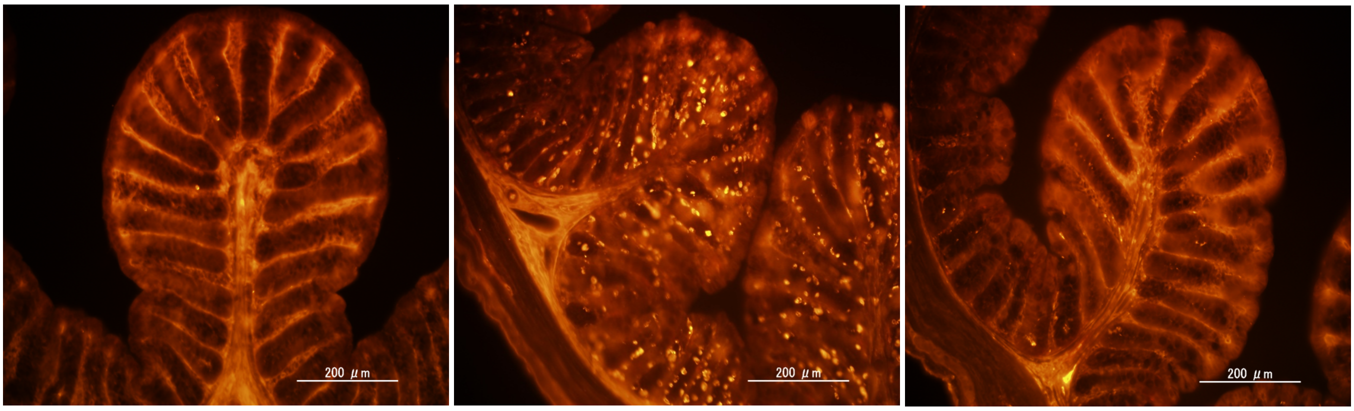

Supplement: Supplementary file 1 — Supplemental Figure 1: Experimental protocol. Method of FA induction and guidance method of oral tolerance in BALB/c mice. Supplemental Figure 2: Occurrence of allergic diarrhea and effects of prior OVA exposure to induction of FA in BALB/c mice Repeated oral OVA (50 mg) challenges resulted in allergic diarrhea in the FA group (■). The occurrence of OVA-induced diarrhea was completely suppressed by prior exposure to 1% OVA in the drinking water for 5 days in the OT–FA group (□). Data are shown as the means ± SE (4 independent experiments, total n = 47–52). ∗∗ p < 0.01 compared with the FA group. Supplemental Figure 3: Effects of the induction of oral tolerance on OVA–specific IgE in the plasma of mice with FA. OVA–specific IgE titers in plasma were markedly increased in FA mice, whereas OVA-specific IgE titers were undetectable in the plasma of OT–FA mice. Data are shown as the means and individual data points. N.D.= not detectable. ++p < 0.01 compared with naïve mice, ∗∗p < 0.01 compared with FA mice, n = 3-4. Supplemental Figure 4: Effects of the induction of oral tolerance on the mRNA expression levels of Th1 and Th2 cytokines in the proximal colons of mice with FA. Th2 cytokine (IL–4) mRNA was significantly up–regulated in FA mice, whereas prior OVA exposure to induction of FA completely prevented the enhanced expression levels of these cytokines. In contrast, the mRNA expression levels of Th1 cytokines (IFN–γ) were nearly identical among the three mice groups. Data are shown as the means and individual data points. ∗∗p < 0.01 compared with naive mice, ++p < 0.01 compared with FA mice, n = 5. Supplemental Figure 5: Effects of the induction of oral tolerance on mMCP–1–positive mucosal mast cells in the proximal colon. The number of mMCP–1 positive mucosal mast cells increased in FA mice. In contrast, OT–FA mice had few mMCP–1 positive mucosal mast cells in their proximal colons. Scale bar = 200 μm. [file 721085.f1.pdf]
